# Anti-tumor activity of a T-helper 1 multiantigen vaccine in a murine model of prostate cancer

**DOI:** 10.1038/s41598-022-17950-1

**Published:** 2022-08-10

**Authors:** Denise L. Cecil, Benjamin Curtis, Ekram Gad, Michael Gormley, Andrew E. Timms, Lauren Corulli, Rinke Bos, Rajendra N. Damle, Manuel A. Sepulveda, Mary L. Disis

**Affiliations:** 1 Cancer Vaccine Institute, University of Washington, 850 Republican Street, Brotman Bld., 2nd Floor, Box 358050, Seattle, WA 98195-8050 USA; 2 Janssen Research and Development LLC, Spring House, PA USA; 3 Janssen Vaccines and Prevention, Leiden, The Netherlands

**Keywords:** Cancer models, Tumour immunology

## Abstract

Prostate cancer is one of the few malignancies that includes vaccination as a treatment modality. Elements of an effective cancer vaccine should include the ability to elicit a Type I T-cell response and target multiple antigenic proteins expressed early in the disease. Using existing gene datasets encompassing normal prostate tissue and tumors with Gleason Score ≤ 6 and ≥ 8, 10 genes were identified that were upregulated and conserved in prostate cancer regardless of the aggressiveness of disease. These genes encoded proteins also expressed in prostatic intraepithelial neoplasia. Putative Class II epitopes derived from these proteins were predicted by a combination of algorithms and, using human peripheral blood, epitopes which selectively elicited IFN-γ or IL-10 dominant antigen specific cytokine secretion were determined. Th1 selective epitopes were identified for eight antigens. Epitopes from three antigens elicited Th1 dominant immunity in mice; PSMA, HPN, and AMACR. Each single antigen vaccine demonstrated significant anti-tumor activity inhibiting growth of implanted Myc-Cap cells after immunization as compared to control. Immunization with the combination of antigens, however, was superior to each alone in controlling tumor growth. When vaccination occurred simultaneously to tumor implant, multiantigen immunized mice had significantly smaller tumors than controls (p = 0.002) and a significantly improved overall survival (p = 0.0006). This multiantigen vaccine shows anti-tumor activity in a murine model of prostate cancer.

## Introduction

Prostate cancer represents the only non-virally mediated solid tumor where a vaccine has been approved for therapeutic use. Sipuleucel-T, a vaccine targeting prostatic acid phosphatase, was shown to prolong overall survival in selected patients with metastatic castration resistant prostate cancer^1^. Patients who were randomized to receive the vaccine had a 4.1-month improvement in overall survival as compared to the placebo control group. The disadvantages of Sipuleucel-T are the complex manufacturing as an autologous cell-based vaccine and the targeting of a single protein expressed in the malignancy. Over the last decade, additional single vaccines have progressed to advanced stage clinical trials but have not demonstrated therapeutic efficacy^2^.

The most common immunogenic proteins in prostate cancer are nonmutated proteins that are aberrantly expressed but weakly immunogenic. Vaccines directed against nonmutated antigens can be associated with the development of Type II immune responses which limit the generation of Type I T-cells via secretion of cytokines such as IL-10^3^. Mutated tumor antigens may be more immunogenic and generate the Type I immune responses needed for an anti-cancer response. Prostate cancers, however, have a low mutation rate with the tumor mutational burden falling well below the threshold needed to generate numerous neoantigens^4^. Microsatellite instability-high and tumor mutational burden high prostate cancer occurs in only 3% of patients^5^. Vaccine approaches designed to stimulate Type I immunity against nonmutated antigens are needed to advance the development of prostate cancer vaccines.

Many vaccines currently under development target a single antigen. Immunogenic proteins commonly used in prostate cancer vaccines include prostatic acid phosphatase and prostate specific antigen that are expressed in the majority of tumors with increased expression as tumor burden increases. There have been few studies aimed to further identify immunogenic proteins that may be expressed earlier in prostate cancer oncogenesis. We have published that antigens expressed early in the progression of malignancy are more likely to be functionally important for tumor growth than those expressed at later stages^6^. Vaccines using early antigens as immunogens were clinically superior to those using antigens expressed later in the course of disease.

Our group has found that within the protein sequence of nonmutated tumor associated antigens are Class II epitopes that selectively elicit either an IFN-γ or an IL-10 dominant response to antigen stimulation. Eliminating IL-10 selective epitopes from an antigen specific vaccine significantly improves clinical efficacy^7^. Further, we have used gene expression data to identify candidate antigens that may be upregulated early and conserved throughout cancer progression^8^. We demonstrate here the identification of Th1 selective epitopes from multiple proteins that are candidates to be upregulated in any invasive prostate cancer regardless of disease histology and develop a multiple antigen vaccine that shows anti-tumor activity in the disease.

## Materials and methods

### Candidate antigen identification

We evaluated gene expression datasets with normal prostate tissue and prostate cancer samples from public repositories and commercial sources to identify genes that were upregulated in both Gleason score ≤ 6 and Gleason score ≥ 8 relative to normal prostate tissue^9,10^. Eight datasets totaling 431 non-cancerous prostate, 143 Gleason score ≤ 6, and 234 Gleason score ≥ 8 prostate cancer samples were analyzed (Supplementary Table S1). Raw expression data in the form of .cel files was normalized using RMA^11^ and the alternative BrainArray CDFs^12^ and combined into a single dataset for analysis. Batch effects observed from combining datasets were removed using COMBAT^13^. Two datasets of 24 normal prostate and 82 prostate cancer samples with undocumented Gleason scores were used to validate gene expression in malignancy. Genes that were upregulated in both lower and higher Gleason score prostate cancer relative to normal were identified using LIMMA^14^. Upregulation was defined as a log2 fold change greater than 1 and false discovery rate less than 0.05.

### Analysis of epitope-specific T-cell responses

Peripheral blood mononuclear cells (PBMC) from 10 volunteer controls were collected and cryopreserved as previously described^15^. Sample collection, after informed consent from all subjects, was carried out in accordance with the relevant guidelines and regulations approved by the University of Washington Human Subjects Division. Peptides, predicted to promiscuously bind human MHC II, were selected with multiple web-based algorithms using our published methods^16^. The peptides were constructed and purified by high-performance liquid chromatography (> 90% pure; CPC Scientific). Human PBMC were evaluated by ELISPOT for antigen-specific IFN-gamma (γ) or IL-10 production as previously described using 10 µg/ml peptides^7^. Mouse splenic cells were evaluated by ELISPOT for antigen-specific cytokine secretion as published, with the following modifications; splenic cells were incubated with antigens for 72 h and spots were developed with the AEC substrate kit (BD Biosciences)^16^. Peptide specific T-cells were documented to respond to protein processed and presented by autologous antigen presenting cells via short term T-cell culture^17^. Positive responses were defined by a statistically significant difference (p < 0.05) between the mean number of spots in the experimental wells and the mean number from no antigen control wells, all performed with 4 replicates. Data are reported as the mean number of spots for each experimental antigen minus the mean number of spots detected in no antigen control wells ± SEM or SD (corrected spots per well: CSPW per 2 × 10^5^ PBMC or 4.5 × 10^5^ splenic cells).

The ratio of the product of the mean magnitude and percent incidence of response was calculated from the IFN-γ and IL-10 ELISPOT assays (IFN-γ^incidence × magnitude^/IL-10^incidence × magnitude^)^18^. Ratios greater than 4 and with ≤ 10% donors generating an IL-10 response defined Th1-selective epitopes and ratios less than 1 defined Th2-selective epitopes. If the ratio was between these two values, the epitope was considered to have a mixed Th1 and Th2 response.

### Animal models and syngeneic tumor cell line

Animal care and use were in accordance with the University of Washington Institutional Care and Use Committee guidelines. Male FVB/N (10 weeks old; median weight: 26 g, range: 23.8–28.5 g; Jackson Laboratory) were used in immunogenicity studies and castrated male FVB/N (7 weeks old; median weight: 24.5 g, range: 20.3–26.9 g; Jackson Laboratory) were used in tumor challenge studies. The mouse cell line Myc-Cap (ATCC) was derived from a spontaneous prostate tumor from Hi-Myc transgenic mice. The cell line was confirmed to be mycoplasma negative and authenticated before use by verification of expression of c-Myc by Western blot.

### Study design

A power analysis was used to detect a difference in tumor volume. Eighty percent or 90% power to detect a significant pairwise difference at the two-sided alpha level of 0.05 was determined to be with 10 or 15 mice per group, respectively. Individual mice were randomized into treatment groups by age and assigned sequentially until the study was fully enrolled. Vaccinated groups were compared to mice injected with adjuvant only. The investigators and animal caretakers were not blinded to the treatment groups, but the treatments were administered in random cage order. Studies were terminated when the volume of the vaccinated group was statistically significantly less than the control for at least two measurements. All mice in in vivo experiments were included in the data analysis. All methods are reported in accordance with ARRIVE guidelines for animal studies.

### Protein expression in tumor cells

Cell lysates derived from Myc-Cap cells and appropriate tissue or cell line controls were separated by SDS/PAGE^19^ and Western blot performed. Antibodies used were mouse anti-PSMA (clone: OTI2E1; 2 µg/mL; Thermo Fisher), rabbit anti-HPN (1 µg/mL; OriGene), mouse anti-AMACR (clone: OTI5F10; 2 µg/mL; Novus Biologicals) rabbit anti-FASN (diluted 1:1000; Cell Signaling Technology), rabbit anti-PSGR (diluted 1:500; Novus Biologicals), rabbit anti-AGR2 (clone: D9V2F; diluted 1:500; Cell Signaling Technology), rabbit anti-ERG (clone: EPR3864; diluted 1:1000; abcam), rabbit anti-CRISP3 (clone: D-6; diluted 1:100; abcam) and HRP-conjugated goat anti-rabbit and rabbit anti-mouse (diluted 1:10,000; Invitrogen).

### Vaccination and assessment of tumor growth

Mice were immunized subcutaneously using a 261/2 G needle. Each mouse was injected with 50 µl of a peptide pool (100 μg each) as a mixture in complete Freund’s adjuvant/incomplete Freund’s adjuvant (Sigma). Four immunizations were given two weeks apart^18^. In the prophylactic vaccine studies, mice were injected with a pool of AMACR-p22/p122/p218 or PSMA-p466/p570/p582 or HPN-p393 or a combination of all epitopes (multiantigen vaccine) and two weeks after the last vaccine, the syngeneic mouse cell line (Myc-Cap; 0.5 × 10^6^ cells) was implanted subcutaneously (n = 10/group)^7^. In some experiments, mice were implanted with Myc-Cap cells, then injected with the first dose of the multiantigen vaccine 24 h hours later (n = 15/group). Mice were terminated when the tumor volume reached 2000 mm^3^ and the whole study examined for survival when the mice reached 115 days of age. Tumors were measured every 2–3 days with Vernier calipers, and tumor volume was calculated as the product of length × width × height × 0.5236. All tumor growth is presented as mean tumor volume (mm^3^ ± SEM).

### Statistical analysis

Model assumptions were checked using the Shapiro–Wilk normality test and by visual inspection of residual and fitted value plots. The unpaired, two-tailed Student’s t-test and One-way ANOVA with Tukey’s Post-Hoc test was used to evaluate differences when normality was confirmed. When normality of the data was not confirmed, the non-parametric Kruskal–Wallis and Man-Whitney tests were used. Differences in tumor volume was determined by two-way ANOVA with a Dunnett post-test for multiple comparisons. Fisher’s exact test was used to evaluate differences between proportions. A p < 0.05 was considered significant (GraphPad Software, Prism v.8).

### Ethical approval

Animal care and use were in accordance with the guidelines approved by the University of Washington Institutional Care and Use Committee.

## Results

### Genes upregulated in both low and high Gleason score samples as well as prostatic intraepithelial neoplasia (PIN) can be identified

**Figure 1:**
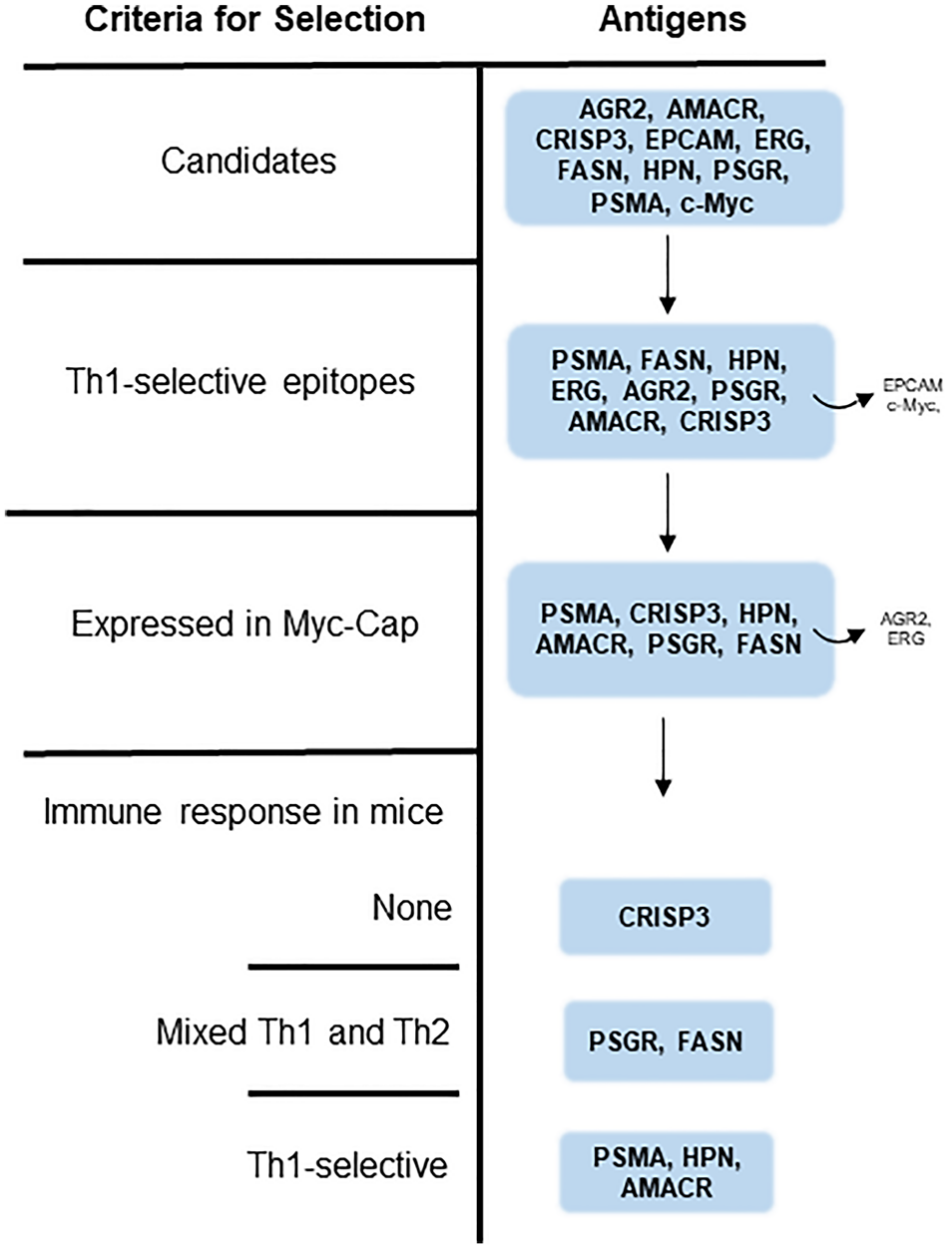
Flow diagram describing multiantigen prostate cancer vaccine antigen selection.

Interrogating a variety of available datasets to select potential antigens expressed early in the development of prostate cancer (Supplementary Table S1), we identified 21 differentially expressed genes in samples from Gleason score (GS) ≤ 6 prostate cancer as compared to non-cancerous tissue. Expression levels of 16/21 genes were either upregulated or further amplified in samples from GS ≥ 8 prostate cancer compared to normal prostate tissue (Supplementary Table S2). We further focused the number of candidates by determining if any were also reported to be upregulated or their corresponding protein overexpressed in PIN. Of the 21 genes, AGR2^20^, AMACR^21^, CRISP3^22^, EPCAM^23^, ERG^24^, FASN^25^, HPN^26^, and OR51E2 (PSGR)^27^ showed some incidence of expression in PIN. We included two additional candidates; PSMA, as this protein is one of the most common vaccine targets for prostate cancer and is expressed in PIN as well as invasive prostate cancer^28^ and c-Myc, as our murine prostate cancer cell line was derived from the Hi-myc transgenic mouse and constitutively expresses c-Myc^29^ (Fig. 1). We then assessed whether Type I T-cells specific for any candidate could be identified in human PBMC.

### Th1 selective epitopes can be identified for many prostate cancer associated proteins

Immunogenicity of the candidates was ascertained by the identification of T-cells which secreted Type I (IFN-γ) or Type II (IL-10) cytokines in response to antigen stimulation of human PBMC^7,18^. Epitopes derived from the prostate cancer associated proteins which elicited a statistically significant response by secretion of either cytokine are shown in Supplementary Figure S1. We hypothesized that the most effective epitopes to include in a vaccine would be those that elicited the highest level of IFN-γ secretion in the greatest number of patients. Overall, there was a significantly greater mean magnitude IL-10 response as compared to the mean magnitude IFN-γ response across all epitopes tested (p < 0.0001), however, Th1 selective epitopes could be identified for several antigens. Three epitopes in PSMA (p466, p570 and p582) were identified as Th1-selective. Two epitopes derived from FASN (p464 and p542) were identified as Th1-selective. Only one HPN epitope (p393) showed selective IFN-γ secretion. Three Th1 selective epitopes could be identified for ERG (p159, p318 and p371), two epitopes were in AGR2 (p6 and p126) and one in PSGR (p97). Three epitopes in AMACR (p22, p122 and p218) were Th1-selective, as were three epitopes in CRISP3 (p138, p163 and p213). All the epitopes predicted for EPCAM or c-Myc induced only IL-10 secretion. The ability of peptide specific T-cells to respond to protein was validated for each six of the eight antigens (Supplementary Fig. S2).

### A subunit vaccine, containing only Th1-selective epitopes, is necessary to inhibit prostate cancer growth

**Figure 2:**
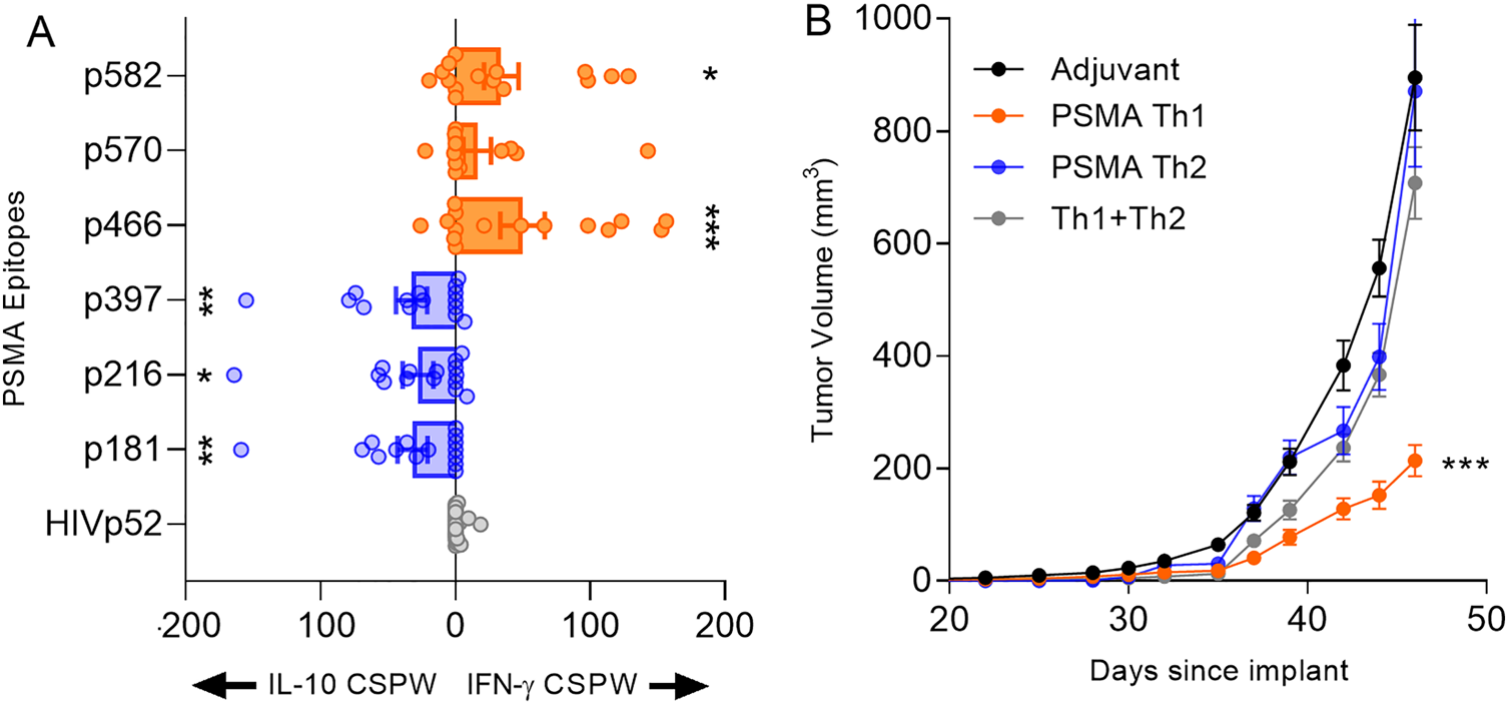
A subunit vaccine, containing only Th1-selective epitopes, is necessary to inhibit prostate tumor growth. (**A**) Mean (± SEM) corrected spots per well (CSPW) for IFN-γ (orange) and IL-10 (blue) in mice vaccinated with the peptide pool PSMA-p397/p216/p181 or PSMA-p582/p570/p466. HIVp52 is used as a negative control peptide. n = 7–8 mice/group; *p < 0.05, **p < 0.01, ***p < 0.001. (**B**) Mean (± SEM) tumor volume (mm3) from mice immunized with the adjuvant alone or the peptide pool PSMA-p582/p570/p466 (PSMA Th1 vaccine) or the peptide pool PSMA-p397/p216/p181 (PSMA Th2 vaccine) or the combination of both vaccines (Th1 + Th2). n = 15 mice/group; ***p < 0.001.

We have previously shown, in a breast cancer model, that vaccines directed against nonmutated overexpressed cancer associated proteins were not effective if IL-10 secreting epitopes were included in the vaccine construct^7^. To validate the observation in prostate cancer, we vaccinated mice with epitopes derived from PSMA and identified three Th1 selective epitopes that secreted significantly higher IFN-γ than IL-10 (p466 (p < 0.0001), p570 (p = 0.06) and p582 (p < 0.0001)) and three epitopes (p181, p216, and p397 (all p < 0.002)) that primarily induced IL-10 secretion as compared to IFN-γ (Fig. 2A). We confirmed PSMA expression in the Myc-Cap cell line. Vaccination with the PSMA Th1-selective epitopes limited tumor growth. The mean tumor volume of PSMA Th1-selective epitope vaccinated mice (214 ± 5.1 mm^3^) was significantly less than that observed in the adjuvant vaccinated control (942 ± 192 mm^3^; p = 0.0006; Fig. 2B). Conversely, the mean tumor volume in mice receiving a PSMA Th2-selective vaccine was no different than the volume observed in the control mice (871 ± 140 mm^3^; p = 0.99). Furthermore, immunizing mice with the Th2-selective epitopes admixed with the Th1-selective epitopes abrogated the anti-tumor effect of the Th1 epitopes. The mean tumor volume of PSMA Th1- and Th2-selective epitope combined vaccinated mice (625 ± 104 mm^3^) was significantly greater than that observed in mice receiving the Th1-selective vaccine (p = 0.0002) but not significantly different than the Th2 vaccine (p = 0.6468) or the adjuvant control (p = 0.3683; Fig. 2B). Thus, a Th1 selective vaccine is needed for prostate cancer inhibition.

### A multiantigen vaccine is more effective than single antigen vaccines in inhibiting prostate cancer growth

**Figure 3:**
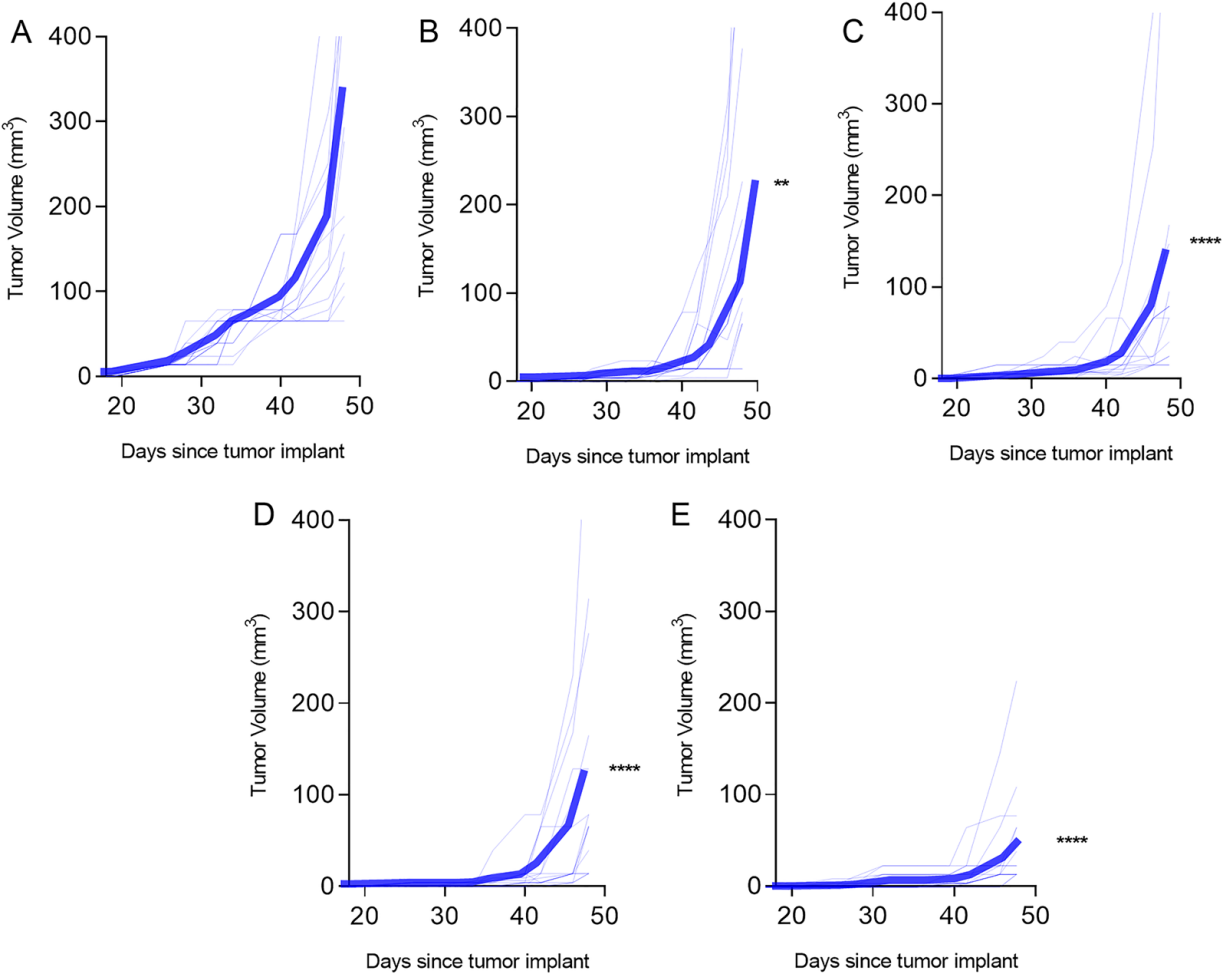
A Th1 selective multiantigen vaccine, administered prior to tumor implant, is effective in inhibiting prostate cancer growth. Tumor volume (mm^3^) from mice vaccinated with (**A**) adjuvant alone or the peptide pools derived from (**B**) AMACR, (**C**) PSMA, (**D**) HPN or (**E**) a combination of the epitopes (multiantigen vaccine). Volume for individual mice (n = 15/group) is presented in thin lines and the mean volume is presented as the thick line. **p < 0.01; ****p < 0.0001.

Of the remaining candidate antigens, AGR2 and ERG were not expressed in the Myc-Cap cell line, therefore were not considered further for in vivo validation (Fig. 1). We vaccinated mice with epitopes from the antigens that were expressed by Myc-Cap cells and in which we identified Th1 selective epitopes (PSMA, HPN, and AMACR; Fig. 1). Additionally we confirmed that the epitopes were highly homologous between mouse and human (PSMA: 93–94%, AMACR: 88–100% and HPN: 88% identical). When used individually, each of the single antigen vaccines demonstrated significant anti-tumor activity. The mean tumor volume from mice immunized with adjuvant alone (314 ± 63 mm^3^; Fig. 3A) was significantly greater than the mean volume from mice immunized with the AMACR vaccine, p218 and p22, (224 ± 60 mm^3^; p = 0.002; Fig. 3B), the PSMA vaccine (141 ± 51 mm^3^; p < 0.0001; Fig. 3C), or the HPN vaccine, p393, (125 ± 38 mm^3^; p < 0.0001; Fig. 3D). When combined into a multiple antigen vaccine, the combination of Th1 selective epitopes could inhibit tumor growth by 84% as compared to control (50 ± 15 mm^3^; p < 0.0001; Fig. 3E). Furthermore, the tumor volume of the mice that received the multiantigen vaccine was significantly lower compared to the mice receiving the AMACR (p < 0.0001), HPN (p = 0.019) or the PSMA (p = 0.002) single antigen vaccines.

**Figure 4:**
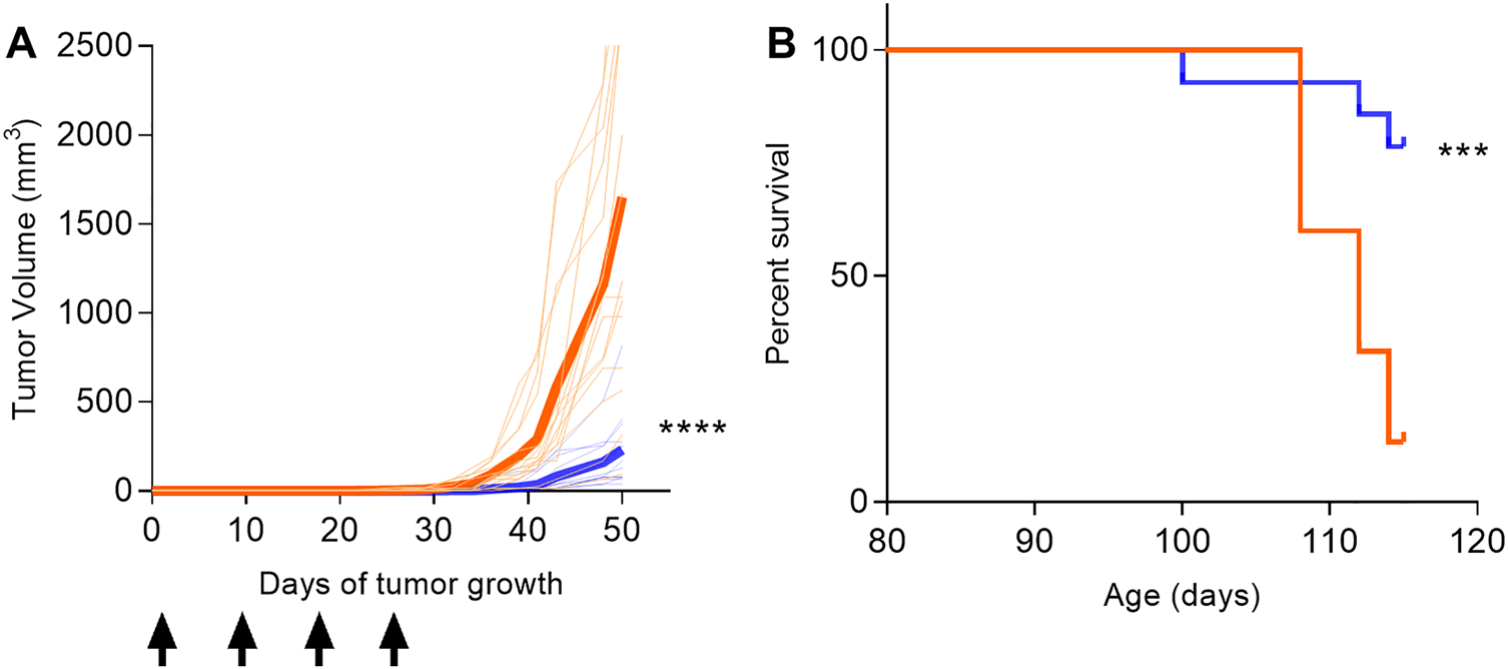
The Th1 selective multiantigen vaccine, administered after tumor implant, limits prostate cancer growth and increases overall survival. (**A**) Tumor volume from mice immunized with adjuvant alone (orange lines) or the multi-antigen vaccine (blue lines). Volume for individual mice (n = 15/group) is presented in thin lines and the mean volume is presented as the thick line. Arrows indicate when the vaccines were administered. (**B**) Kaplan Meir curve for percent survival from mice immunized with adjuvant alone (orange line) or the multiantigen (blue line). n = 15 mice/group; ***p < 0.01, ****p < 0.0001.

We immunized mice after Myc-Cap cells were implanted. The mean tumor volume from mice immunized with adjuvant alone (1650 ± 293 mm^3^) was significantly greater than the mean volume from mice immunized with the multiantigen vaccine 50 days after Myc-Cap implant (231 ± 55 mm^3^; p = 0.002; Fig. 4A). In a survival study, 79% of vaccinated mice were alive at 115 days as compared to 13% of the control mice (p = 0.0006; Fig. 4B).

## Discussion

Phase II and III clinical trials of single antigen prostate cancer vaccines show promise in disease modulation and provide a foundation for the further development of effective vaccination approaches including optimizing antigen choice and vaccine technology. Data presented here demonstrate that antigens can be identified that are present early in prostate cancer development. These antigens are overexpressed in PIN and expression is maintained through invasive prostate cancer. We also show vaccines targeting nonmutated prostate cancer associated proteins inhibit tumor growth only if IL-10 inducing epitopes are edited from the vaccine. Finally, data suggests that a multiantigen Th1 selective vaccine targeting proteins expressed throughout the progression of prostate cancer may have anti-tumor effects.

Many prostate cancer antigens are chosen for vaccination due to the incidence of expression of that protein in the majority of tumors. Three of the most common immunologic targets, prostate specific antigen, prostatic acid phosphatase, and prostate specific membrane antigen are highly expressed in nearly all prostate cancers with increasing expression in advanced stage disease and metastases^30–32^. To identify early antigens in prostate cancer we analyzed gene expression data including samples that represented tumors with a span of Gleason scores (Supplementary Table S1) both less than 6 and greater than 8 and chose upregulated genes that were common to both groups. We have used this approach to identify genes encoding immunogenic proteins that are overexpressed in colon adenomas through invasive colon cancers^8^. Similar to the study cited above, the genes encoding conserved overexpressed colon cancer associated proteins were functionally relevant. Silencing these genes significantly reduced proliferation in tumor cell lines representing a variety colon cancer subtypes and induced apoptosis of both adenoma and colon cancer cell lines^8^. Further, we were able to discern our vaccine antigens were overexpressed in PIN. Although PSMA is highly expressed in both primary invasive prostate cancer and metastasis^28^, the protein is also overexpressed in about 50% of PIN samples studied^33^. Expression of HPN is strongest in PIN and still highly expressed in primary prostate cancer with expression receding in metastatic disease^26^. Overexpression of AMACR occurs in the majority of prostate cancers as well as 90% (125/140) of cases of high-grade PIN^21,34^. Similar to the early antigens identified for breast and colon cancer, silencing gene expression of either HPN or AMACR significantly inhibits prostate cancer cell growth^35,36^. Immunologically targeting functionally important proteins should improve prostate cancer vaccine efficacy.

Type I immunity is needed for cancer eradication. Type I tumor infiltrating lymphocytes have been associated with an improved prognosis in prostate cancer^37^. Unfortunately, the immune microenvironment in prostate cancer is dominated by Type II and regulatory T-cells. Tumor infiltrating T-regulatory cells are associated with both higher Gleason score tumors as well as larger tumor size^38^. Moreover, the metabolites produced by innate immune cells associated with chronic prostate inflammation inhibit the development and proliferation of Tbet + Type I CD4 T-cells^39^. A pitfall of vaccines targeting nonmutated tumor antigens is the potential induction of epitope specific T-regulatory cells. Studies have shown immunization with NY-ESO in melanoma, for example, can increase both existing and newly induced T-regulatory cells specific for epitopes within the vaccine^40^. In prostate cancer, the presence of T-regulatory cells has been shown to inhibit the patient’s endogenous prostate antigen specific immune response^41^. Vaccine strategies designed to increase T-helper 1 immunity can alter the tumor microenvironment. Type I cytokines secreted by CD4 T-cells homing to tumor will enhance the cross-priming of CD8 T-cells^42^. Furthermore, IFN-γ secreted by vaccine primed Type I CD4 T-cells can have antitumor effects by indirectly inhibiting growth factor receptor signaling^43^. Our group has found within the sequence of nonmutated tumor associated proteins are class II epitopes that can selectively induce either an IFN-γ dominant or IL-10 dominant response^44^. Unless the epitopes associated with IL-10 secretion are edited from the vaccine the anti-tumor effect of the vaccine will be limited^7^. We have recapitulated that observation in prostate cancer using PSMA epitope-based immunization as an example (Fig. 3). Although the majority of epitopes derived from the prostate cancer antigens we identified induced IL-10 secretion, we were able to identify Th1 selective epitopes from eight of the proteins. These data suggest that Th1 selective multiantigen vaccines suitable for immunization of prostate cancer patients can be developed for nonmutated tumor antigens.

Clinical trials of prostate cancer vaccines have focused on immunization with single antigens. While all our single antigen vaccines all demonstrated significant anti-tumor activity, the combination of antigens into one vaccine showed profound inhibition of prostate cancer growth and was superior to any single antigen vaccine. We have shown multiantigen vaccines are superior to single antigen approaches in other models^45^. Multiantigen immunization, prior to Myc-Cap implant, could inhibit the development of prostate cancer by 86% as compared to control. Multiantigen vaccine immunized mice had significantly lower tumor burden and an improved overall survival as compared to control. There are limitations to this study. One concern is that a strong Th1 response will induce self-regulation over time and we were not able to evaluate the tumor microenvironment in our survival experiments. In a Phase II trial of Sipuleucel-T focused on biomarkers, while Th1 and cytotoxic T-cells could be identified in patients’ tumors after vaccination, upregulation of immune checkpoint proteins also occurred^46^. A further limitation to our in vivo experiments is the Myc-Cap model. While inhibition of tumor growth can be a surrogate for disease prevention, ideally, we would be able to test the vaccine in a transgenic mouse model of prostate cancer and demonstrate primary prevention. PIN can be detected as early as two weeks old in Hi-Myc transgenic mice and the disease progresses quickly limiting the ability to vaccinate repeatedly after pups are weaned^29^.

Prostate cancer can be immunologically modulated through vaccination to the clinical benefit of patients, but there is room for improvement. One approach is to combine antigen specific vaccination with administration of an immune checkpoint inhibitor. A recent study evaluated vaccination with Sipuleucel-T followed by treatment with ipilimumab in 50 patients with metastatic castration-resistant prostate cancer (mCRPC). Clinical responses were seen in 12% of treated patients demonstrating modest clinical activity^47^. An additional study in 40 patients with mCRPC vaccinated with a DNA vaccine encoding prostatic acid phosphatase with either sequential or concurrent administration of pembrolizumab^48^. Although there was only one partial response in the group of 25 patients with measurable disease, 10% of patients experienced a greater than 50% decline in PSA levels. Moreover, thirty two percent of patients remained on trial greater than 6 months without disease progression. These data are promising that combination immunotherapeutic approaches may have benefit over single modality therapy in prostate cancer. A better understanding of the role of antigens in mediating the anti-tumor response, multiple antigen vaccines, and newer vaccine technologies and adjuvants may allow the generation of an immune response that can overcome the immunosuppressive environment that develops with prostate cancer progression.

## Conclusion

Multiple antigens can be identified in prostate cancer. Vaccines designed to selectively elicit IFN-Ɣ antigen specific T-cells can be developed and formulated into a multiple antigen, multiple epitope vaccine. Immunization with multiple antigens is more effective in limiting tumor growth than immunization with single antigens alone. These data allow the development of vaccines directed against non-mutated tumor antigens that may be more clinically effective.

## Supplementary Information

Supplementary Information.

## Data Availability

The datasets analyzed during the current study are available in the Gene Expression Omnibus (GEO) repository: ascension numbers GSE32571, GSE40272, GSE41969, GSE29079, GSE26022, GSE32448, GSE3325, GSE6956 or the Array Express repository, ascension number E-TABM-26.
