# Supplementary material for: Anti-tumor activity of a T-helper 1 multiantigen vaccine in a murine model of prostate cancer
Source: Sci Rep. 2022 Aug 10;12:13618. doi: 10.1038/s41598-022-17950-1 (PMC9365795; doi:10.1038/s41598-022-17950-1)
Supplement: Supplementary file 1 — Supplementary Information. [file 41598_2022_17950_MOESM1_ESM.pptx]

## Slide 1
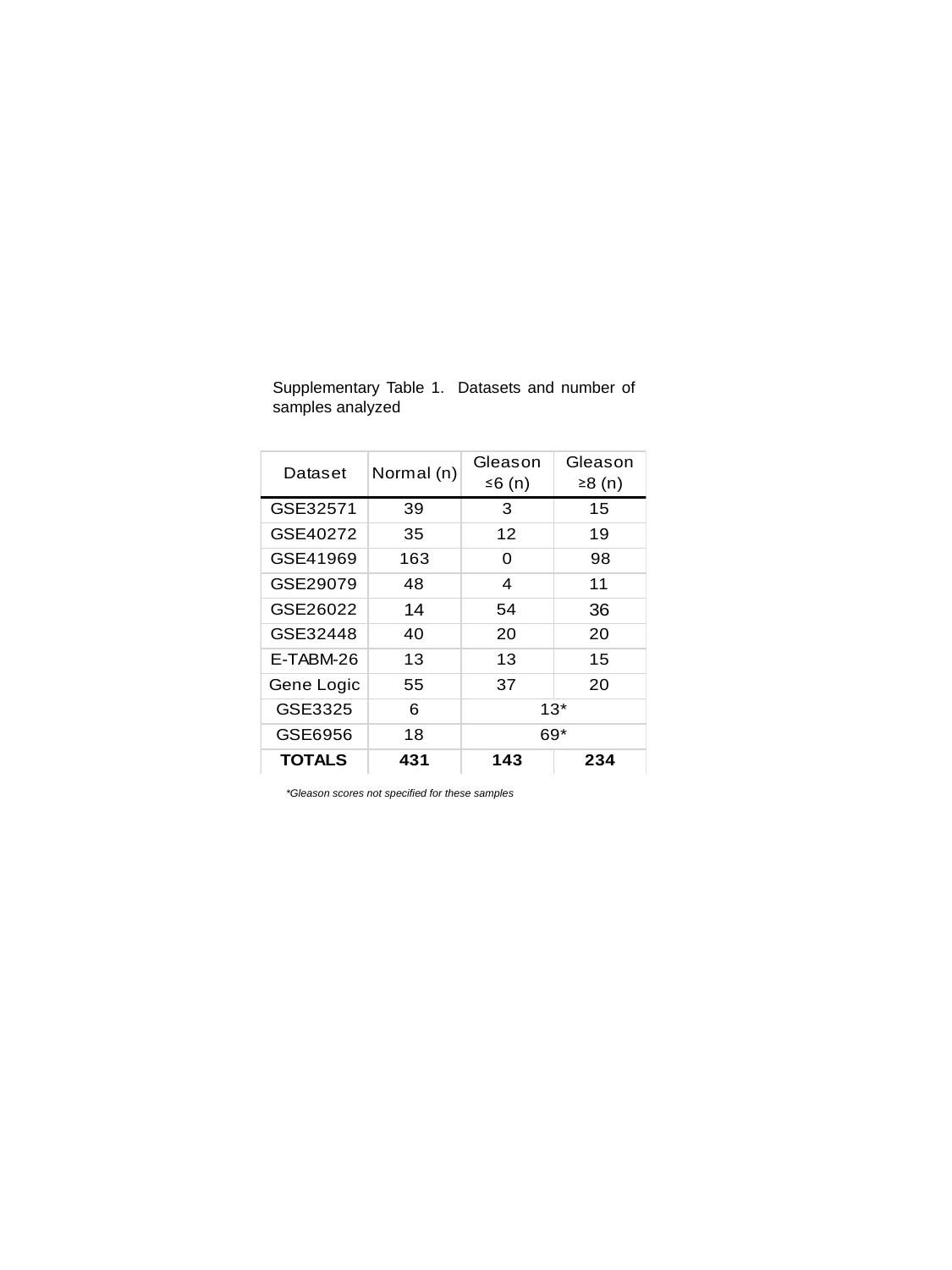

Supplementary Table 1. Datasets and number of samples analyzed
*Gleason scores not specified for these samples

## Slide 2
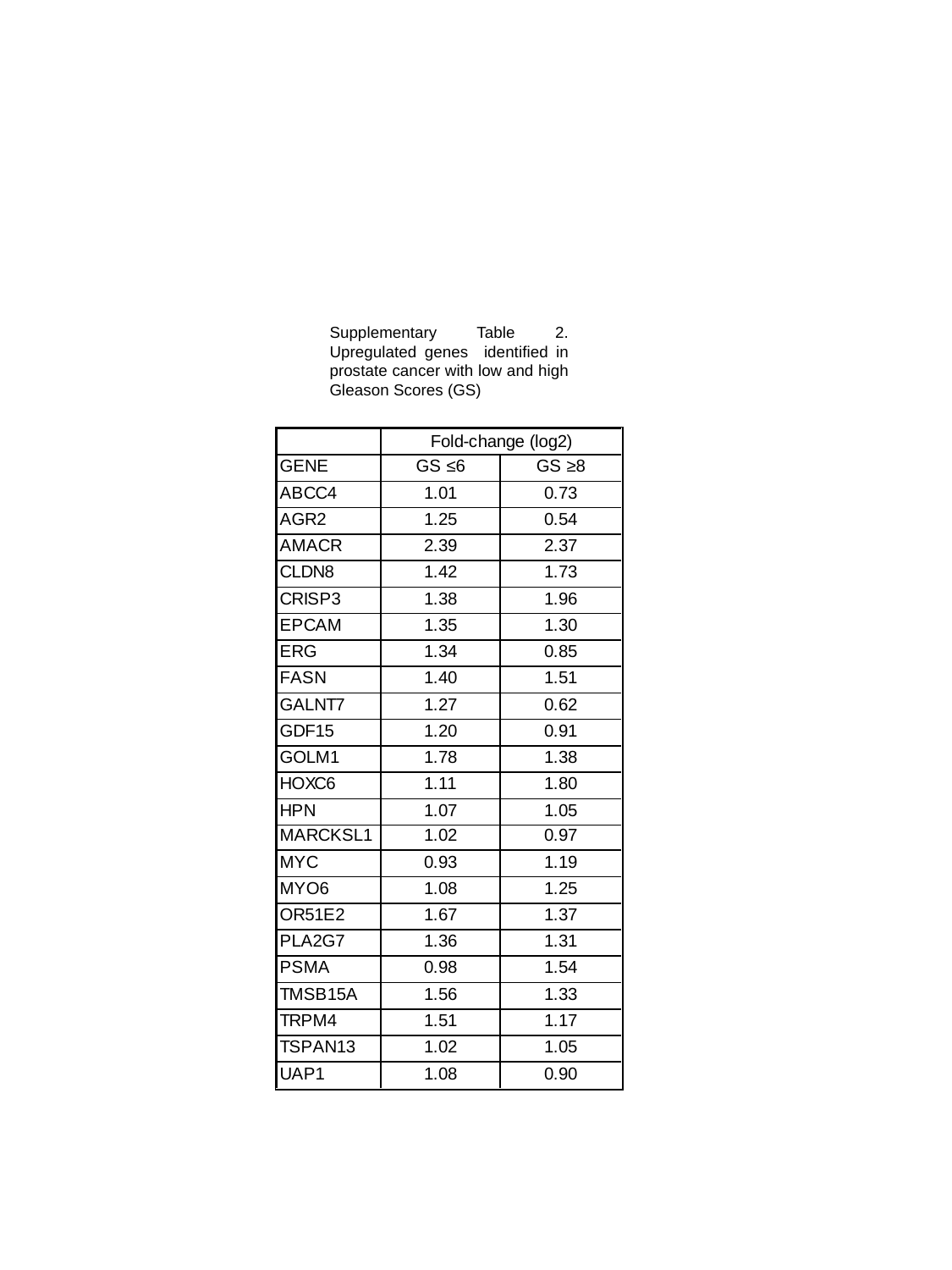

Supplementary Table 2. Upregulated genes identified in prostate cancer with low and high Gleason Scores (GS)

## Slide 3
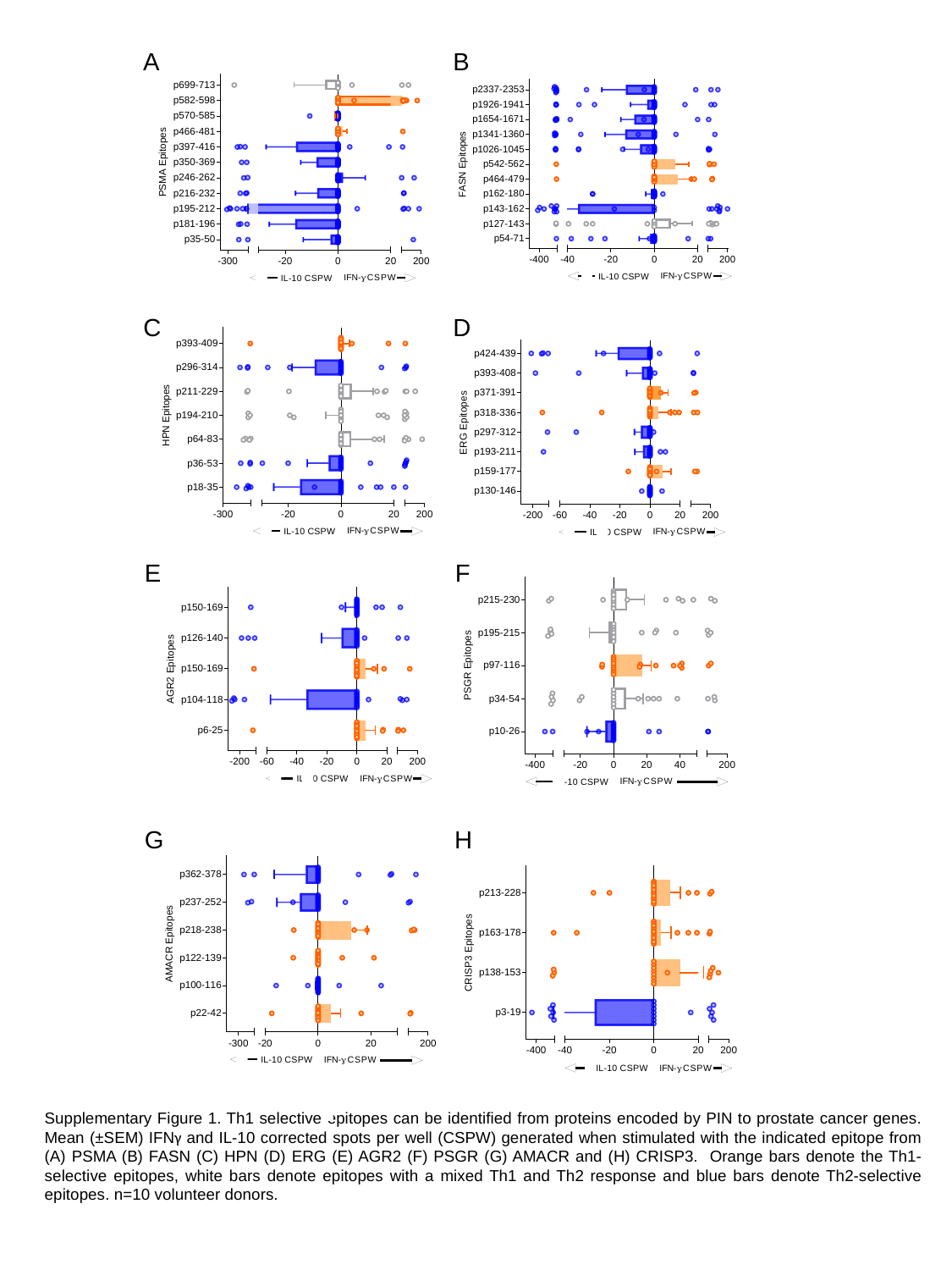

A
B
C
D
E
F
G
H
Supplementary Figure 1. Th1 selective epitopes can be identified from proteins encoded by PIN to prostate cancer genes. Mean (±SEM) IFNγ and IL-10 corrected spots per well (CSPW) generated when stimulated with the indicated epitope from (A) PSMA (B) FASN (C) HPN (D) ERG (E) AGR2 (F) PSGR (G) AMACR and (H) CRISP3. Orange bars denote the Th1-selective epitopes, white bars denote epitopes with a mixed Th1 and Th2 response and blue bars denote Th2-selective epitopes. n=10 volunteer donors.

## Slide 4
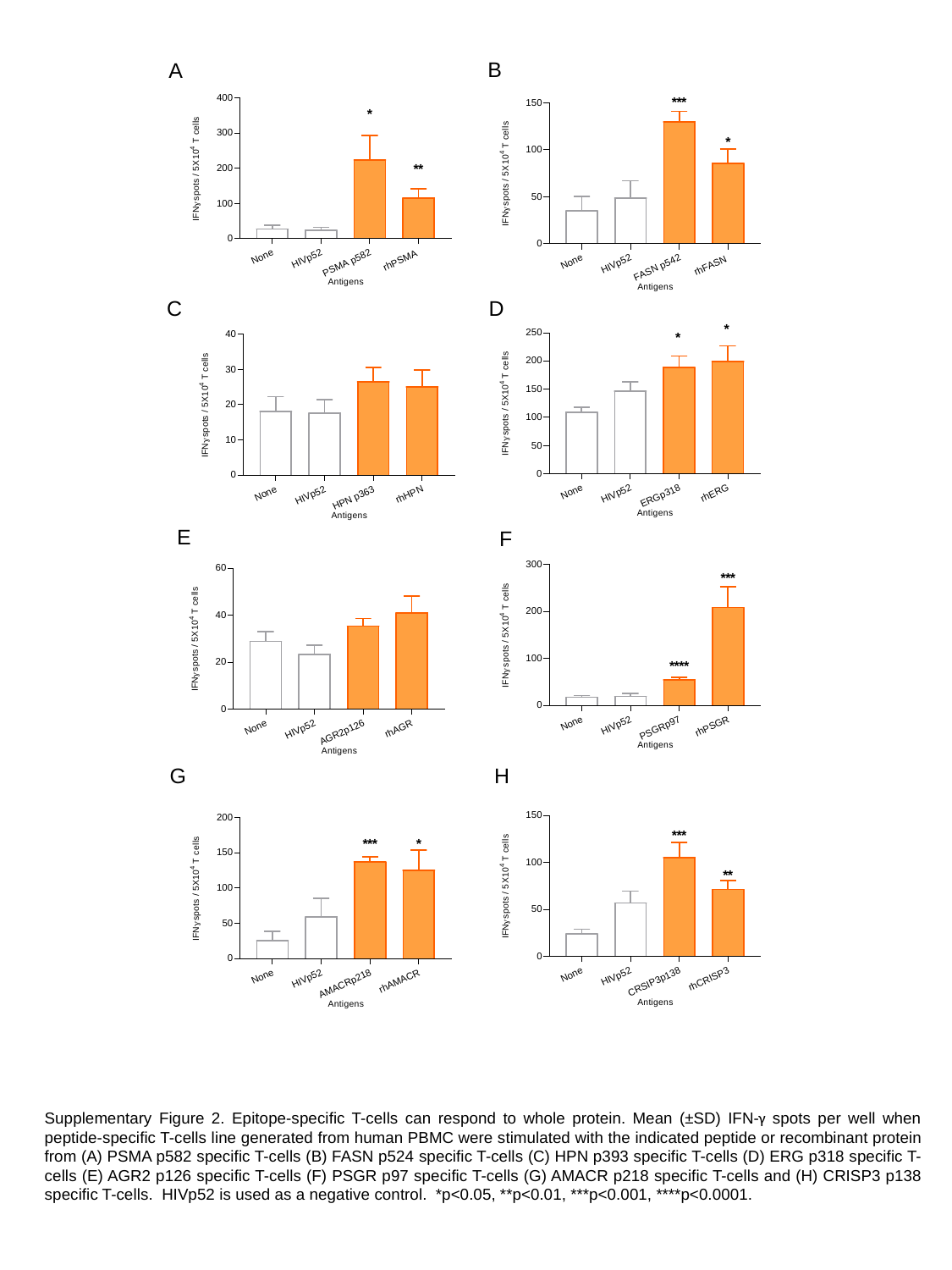

B
A
C
D
E
F
G
H
Supplementary Figure 2. Epitope-specific T-cells can respond to whole protein. Mean (±SD) IFN-γ spots per well when peptide-specific T-cells line generated from human PBMC were stimulated with the indicated peptide or recombinant protein from (A) PSMA p582 specific T-cells (B) FASN p524 specific T-cells (C) HPN p393 specific T-cells (D) ERG p318 specific T-cells (E) AGR2 p126 specific T-cells (F) PSGR p97 specific T-cells (G) AMACR p218 specific T-cells and (H) CRISP3 p138 specific T-cells. HIVp52 is used as a negative control. *p<0.05, **p<0.01, ***p<0.001, ****p<0.0001.
